# Identification and validation of genomic regions influencing kernel zinc and iron in maize

**DOI:** 10.1007/s00122-018-3089-3

**Published:** 2018-03-24

**Authors:** Vemuri Hindu, Natalia Palacios-Rojas, Raman Babu, Willy B. Suwarno, Zerka Rashid, Rayalcheruvu Usha, Gajanan R Saykhedkar, Sudha K. Nair

**Affiliations:** 1 Asia Regional Maize Program, International Maize and Wheat Improvement Center (CIMMYT), ICRISAT Campus, Patancheru, Hyderabad, Telangana 502324 India; 2 International Maize and Wheat Improvement Center (CIMMYT), Km 45 Carretera Mexico-Veracruz, 56130 Texcoco, Mexico; 3 Sri Padmavati Mahila Visvavidyalayam (Women’s University), Tirupati, Andhra Pradesh 517502 India; 4 Present Address: Multi-Crop Research Center (MCRC), DuPont Pioneer, Hyderabad, Telangana 500078 India; 5 Present Address: Department of Agronomy and Horticulture, Faculty of Agriculture, Bogor Agricultural University, Jl. Meranti Kampus IPB Dramaga, Bogor, 16680 Indonesia; 6 Present Address: Project Director, SPMESM, Dr. Hedgewar Hospital, Aurangabad, Maharashtra 431005 India

## Abstract

**Key message:**

Genome-wide association study (GWAS) on 923 maize lines and validation in bi-parental populations identified significant genomic regions for kernel-Zinc and-Iron in maize.

**Abstract:**

Bio-fortification of maize with elevated Zinc (Zn) and Iron (Fe) holds considerable promise for alleviating under-nutrition among the world’s poor. Bio-fortification through molecular breeding could be an economical strategy for developing nutritious maize, and hence in this study, we adopted GWAS to identify markers associated with high kernel-Zn and Fe in maize and subsequently validated marker-trait associations in independent bi-parental populations. For GWAS, we evaluated a diverse maize association mapping panel of 923 inbred lines across three environments and detected trait associations using high-density Single nucleotide polymorphism (SNPs) obtained through genotyping-by-sequencing. Phenotyping trials of the GWAS panel showed high heritability and moderate correlation between kernel-Zn and Fe concentrations. GWAS revealed a total of 46 SNPs (Zn-20 and Fe-26) significantly associated (*P* ≤ 5.03 × 10^−05^) with kernel-Zn and Fe concentrations with some of these associated SNPs located within previously reported QTL intervals for these traits. Three double-haploid (DH) populations were developed using lines identified from the panel that were contrasting for these micronutrients. The DH populations were phenotyped at two environments and were used for validating significant SNPs (*P* ≤ 1 × 10^−03^) based on single marker QTL analysis. Based on this analysis, 11 (Zn) and 11 (Fe) SNPs were found to have significant effect on the trait variance (*P* ≤ 0.01, *R*^2^ ≥ 0.05) in at least one bi-parental population. These findings are being pursued in the kernel-Zn and Fe breeding program, and could hold great value in functional analysis and possible cloning of high-value genes for these traits in maize.

**Electronic supplementary material:**

The online version of this article (10.1007/s00122-018-3089-3) contains supplementary material, which is available to authorized users.

## Introduction

Advances in agricultural research and technology have resulted in the increase of food grain production to meet the needs of an increasing human population. Though the increase in food grain production helped to meet the calorie requirement, low levels of micronutrients, zinc (Zn), iron (Fe), and pro-vitamin A among others, are a global cause for malnutrition related health impairments which could lead to socio-economic losses, reduced work performance and productivity (Diepenbrock and Gore 2015; Tiwari et al. 2016). Micronutrient malnutrition, known as “hidden hunger”, is more prevalent among pregnant women and infants dwelling in the developing world, where people mostly rely on cereal-based diets (Diepenbrock and Gore 2015). In a specific mention about Zimbabwe, Banziger and Long (2000) reported that there was approximately 30% of pregnant and lactating women who are Fe-deficient. Although micronutrients are required in a relatively small quantity for humans, they play a vital role to stimulate cellular growth, signaling, function and metabolism (Guerrant et al. 2000; Kapil and Bhavna 2002). Micronutrients are not synthesized by humans and must be acquired through the diet. Several strategies have been used to enrich human diet with micronutrients, including supplementation, dietary diversity and commercial food fortification. However, implementing such interventions on a large scale in resource-poor communities incur substantial recurring expenditure and are frequently found to be economically unsustainable in the long term (Bouis and Welch 2010). Therefore, ‘bio-fortification’ which refers to micronutrient enrichment of major staple food crops like maize, rice, and wheat through classical and modern plant breeding strategies assume considerable significance (Graham and Welch 1996; Bouis 2000; Welch and Graham 2002; Babu et al. 2013).

Maize alone contributes over 20% of total calories in human diets in 21 countries and over 30% in 12 countries that are home to more than 310 million people (Smale et al. 2013). Bio-fortification of maize is, therefore, expected to improve the health and welfare of many disadvantaged populations across the globe. Significant progress has been made in developing, testing, and deploying bio-fortified maize, especially quality protein maize (QPM) (Atlin et al. 2010) and pro-vitamin A-enriched maize (Pixley 2013) worldwide. Studies have demonstrated that high-Zn maize will be impactful in rural areas with limited access to dietary supplements and fortified foods (Chomba et al. 2015). Recently, a Zn-biofortified maize variety BIO-MZN01 with 36% increased Zn on average than other maize varieties was released in Colombia in 2018. This variety was developed by International Maize and Wheat Improvement Center (CIMMYT) with the support of HarvestPlus in collaboration with the Agriculture for Nutrition Health (A4NH) and International Center for Tropical Agriculture (CIAT) (http://www.HarvestPlus.org).

Based on estimated average requirement (EAR) of 1860 µg/day of Zn and 1460 µg/day of Fe, the target level of Zn and Fe was established as 33 and 52 µg/g, respectively, in maize kernels (Bouis and Welch 2010). The baseline content for Zn in maize is about 20 µg/g, therefore, an increase of 13 µg/g is feasible by conventional breeding, especially because a wide range of Zn concentration is available in the germplasm. However, for Fe, such natural diversity has not been found and an increase of about 30 µg/g might be more attainable using alternate methods such as gene editing or transgenics (Ortiz-Monasterio et al. 2007).

Understanding the extent of genetic variability for kernel micronutrients in elite maize germplasm along with the genetics of accumulation mechanisms will be critical for the development of nutrient-enriched varieties. Kernel micronutrient concentration depends upon a number of factors such as micronutrient availability, environmental conditions, mobilization of nutrients from soil, uptake by roots, translocation, redistribution within the plant, and deposition in the seeds. Each of these processes is likely governed by many genes (Bashir et al. 2012; Kobayashi and Nishizawa 2012). Several genes related to metal transport, phytosiderophore biosynthesis, mineral ion sequestration and grain portioning have been identified in Arabidopsis, rice, wheat, barley, maize, tomato and soybean (Eide et al. 1996; Zhao and Eide 1996; Grotz et al. 1998; Eckardt 2000; Vert et al. 2001; Waters 2002). Sharma and Chauhan (2008) predicted a total of 48 candidate genes to be involved in the Fe and Zn transport in maize based on putative candidate genes, viz., 13 genes from ZIP (zinc-regulated transporter/iron-regulated transporter proteins) family, 16 from NRAMP (natural resistance associated macrophage protein) family, 17 from YS (yellow stripe) family and one each from CE (cation efflux) family and ferritin family. This makes the accumulation of minerals in seeds a complex polygenic phenomenon.

A large number of maize germplasm accessions with high levels of kernel-Zn (3.81–95.62 mg/kg) and Fe (9.6–159.43 mg/kg) have been identified in temperate (Ahmadi et al. 1993; Brkic et al. 2004) and tropical collections (Banziger et al. 2000; Menkir 2008; Chakraborti et al. 2011; Prasanna et al. 2011), in mid-altitude and low-land agro-ecologies including landraces, inbreds, hybrids and open pollinated varieties. Genome analysis tools provide access to thousands of genomic polymorphisms, thus considerably broadening our capacity to monitor and effectively utilize genetic diversity (Glaszmann et al. 2010). Genome-wide association studies (GWAS) based on linkage disequilibrium (LD) is a robust approach for mapping biologically valuable traits in germplasm and has been successfully applied in a range of plant species (Huang and Han 2014; Yang et al. 2014). LD is the non-random association of alleles at different loci, measured as *r*^2^ and *D*′ (Flint-Garcia et al. 2003). Genotyping-by-sequencing (GBS) methodology (Elshire et al. 2011) offers a less expensive method for genotyping large number of samples, and provides around a million SNPs, and hence is extremely relevant to LD-based mapping in a crop like maize which has reported faster LD decay. Validated marker-trait associations from GWAS will be of great value in developing bio-fortified maize with high kernel-Zn and Fe. Objectives of the current study were (1) to assess the genetic variation for kernel-Zn and Fe concentrations in a wide array of maize germplasm; (2) to identify the genomic regions that influence kernel-Zn and Fe concentrations through GWAS and develop high throughput and easy to use SNP assays; and (3) to validate GWAS-identified genomic regions in bi-parental populations.

## Materials and methods

### Plant materials and growing environments

A set of 923 inbred lines representing CIMMYT and partners’ germplasm was used as an association mapping panel. The panel included 432 tropical, 402 subtropical and 89 temperate germplasm. In addition to elite breeding lines, a number of lines from improved pools and populations formed to serve as sources for drought and Low N tolerance were included, as well as lines that were developed for QPM.

This panel was grown in three different environments at CIMMYT research stations in Mexico: at Agua Fria in 2012 (AF12A) and 2013 (AF13A) and at Celaya in 2012 (CE12B). Agua Fria is located at 20°32′N, 97°28′W, 110 m above sea level (masl), and has average annual temperature of 22 °C with average annual precipitation of 1200 mm. Celaya is located at 20°26′N, 103°19′W; 1750 masl; average annual temperature 19 °C; average annual precipitation 700 mm. The trials at AF12A and CE12B were laid out in a randomized complete block design (RCBD) with two replications, whereas at AF13A, in alpha lattice design with two replications. The rows were of 2.5 m length and 75 cm apart and each entry was grown in a single row plot. Two to six plants from each plot were self-pollinated, hand-harvested and hand-shelled to avoid any metal contamination. Kernels were bulked for subsequent micronutrient analyses. Soil samples were also taken in at least five distal points in the field trials and at 0–30 cm deep. Samples were dried and sent for analysis at Fertilab commercial laboratory in Mexico.

From the association mapping panel, three lines with high Zn and three lines with moderate or low Zn lines were selected based on the micronutrient analysis to form the bi-parental populations. Three double-haploid (DH) populations were derived from the crosses between high Zn lines with low or moderate Zn lines (Table 2). The three DH populations (DHP1, DHP2 and DHP3) had population sizes of 96, 112 and 143, respectively. These populations were planted in single replication trials in two environments at Celaya in 2014 and in Tlatizapan, Morelos, Mexico in 2015. Planting conditions and micronutrient analysis methods were the same as described above.

### Micronutrient analysis

Random samples of 6 g were used for analysis. Only six kernels were ground into fine powder (< 0.5 µm), using a Retsch™ miller (model MM400) and 35 mL grinding milling jar of zirconium. Milling time was 90 s at 30 Hz. Flour was collected in 15 mL plastic tubes and analyzed by X-ray fluorescence using X-ray fluorometer (XRF) Oxford instruments™, model X-Supreme 8000^®^. Five grams of flour was placed in the polypropylene capsules and closed with a Poly-4^®^ Oxford Instruments™, and readings were recorded. About 10% of the samples were also analyzed by inductive coupled plasma (ICP) as described by Galicia et al. (2012) to confirm accuracy of values obtained by XRF. In ICP analysis, aluminum and titanium were also monitored as indicators of contamination.

### Genotyping

DNA was extracted from leaf samples of 3–4-week-old seedlings using the standard CIMMYT laboratory protocol (Cimmyt 2005). The association mapping panel and three DH populations under study were genotyped for single nucleotide polymorphism (SNP) using genotyping-by-sequencing (GBS) method at the Institute for Genomic Diversity, Cornell University, Ithaca, NY, USA. Physical coordinates of all SNPs were derived from the maize reference genome version B73 AGPV2. The genotypic data consisted of 955,690 SNPs across all the chromosomes, in the imputed GBS SNP dataset of approximately 22,000 maize samples publicly available through Panzea (http://www.panzea.org). From this, a smaller dataset of 347,765 SNPs which met the filtering criteria of call rate (CR) ≥ 0.7 and minor allele frequency (MAF) ≥ 0.03 was used for GWAS. For principal component and kinship analyses, 69,830 SNPs with filtration criteria of CR ≥ 0.9 and MAF ≥ 0.1 were used.

### Phenotypic data analysis

Variance components, *σ*^2^*G*, *σ*^2^*GE* and *σ*^2^*e*, for the multi-environmental phenotypic data were estimated from analysis of variance (ANOVA) using multi environment trial analysis with R (METAR) (Alvarado et al. 2015). Broad-sense heritability (*H*^2^) of the trails was estimated as:

$$H^{2} = \frac{{\sigma^{2} G}}{{\sigma^{2} G + \sigma^{2} GE /l + \sigma^{2} e /lr}},$$

where *σ*^2^*G* is the genotypic variance, *σ*^2^*GE* is the genotype × environment variance, *σ*^2^*e* is the error variance, *l* is the number of environments, and *r* is the number of replications. Correlation coefficients between environments and traits, summary statistics (mean, SE, range, LSD, CV) were also generated using standard procedures implemented in METAR. Best linear unbiased estimators (BLUEs) used for GWAS was estimated as

$$Y_{ijkl} = \mu + {\text{Loc}}_{l} + {\text{Rep}}_{j} ({\text{Loc}}_{l} ) + {\text{Block}}_{k} ({\text{Rep}}_{j } {\text{Loc}}_{l} ) + {\text{Gen}}_{i} + {\text{Loc}}_{l} \times {\text{Gen}}_{j} + \varepsilon_{ijkl} ,$$

where *Y*_*ijkl*_ is the response value of observed trait, *µ* the overall mean, Gen_*i*_ is the treatment fixed effect (*i* = 1, 2,…, *n*), Rep_*j*_ is the replicate effect (*j* = 1, 2,…, *n*), Block_*k*_ is the block effect, Loc_*l*_ is the location effect and *ε*_*ijkl*_ is the error term.

### Population structure, kinship and genome-wide linkage disequilibrium

The principal component analysis (PCA) method as described by Price et al. (2006), implemented in SNP and Variation Suite (SVS) V_8.6.0 (SVS, Golden Helix, Inc., Bozeman, MT, USA, http://www.goldenhelix.com) was used for the analysis. A three-dimensional plot of the first three principal components was drawn to visualize the possible population stratification among the samples. A kinship matrix was also computed from identity-by-state (IBS) distance matrix as executed in SVS V_8.6.0:

$${\text{IBS}}\,{\text{distance}} = \frac{{{\text{No}} .\,{\text{of}}\,{\text{markers}}\,{\text{IBS2 + (0}} . 5\, \times \,{\text{No}} .\,{\text{of}}\,{\text{markers}}\,{\text{in}}\,{\text{IBS1)}}}}{{{\text{No. of non-missing}}\,{\text{markers}}}},$$

where IBS1 and IBS2 are the states in which the two inbred lines share one or two alleles, respectively, at a marker (Bishop and Williamson 1990).

Linkage disequilibrium (LD) was quantified as adjacent-pairwise *r*^2^ values (the squared allele frequency correlations, among alleles at two adjacent SNP markers) (Hill and Robertson 1968) and was estimated for 34,420 SNPs using SVS V_8.6.0. To investigate the extent of linkage disequilibrium (LD) decay across the genome, *r*^2^ values were plotted against the physical distance within the SNPs (Remington et al. 2001). The ‘nlin’ function in the statistical programming language R (R Core Team 2017) was used to obtain LD decay plot as non-linear model.

### GWAS for kernel-Zn and Fe

For each trait, three different association analyses were carried out: uncorrected (*U*), corrected for population structure (*Q*), and corrected for population structure and kinship (*Q* + *K*) using SVS V_8.6.0. In the uncorrected analysis, associations were tested in an additive model without correcting for any of the confounding variables. In the *Q* model (GLM—general linear model, or fixed-effect linear model), the associations were corrected using population structure through principal component analysis, in which ten principal components (PC) were included. In the *Q* + *K* model (MLM—mixed linear model), associations were corrected using both PCs and kinship matrix. All the three models (*U*, *Q* and *Q* + *K*) involved testing one variant at a time. Manhattan plots were plotted using the − log 10 *P* values of all SNPs used in analysis. The appropriateness of the different models was evaluated through *Q*–*Q* plots that were obtained by plotting ‘expected − log10 *P* values’ on the *x*-axis and ‘observed − log10 *P* values’ on the *y*-axis. Multiple testing correction was performed to determine the significance threshold, where instead of 345,767 independent tests, the total number of tests were estimated based on the average extent of LD at *r*^2^ = 0.1 (Cui et al. 2016). Based on this, significant associations were declared when the *P* values in independent tests are less than 5.03 × 10^−05^ or − log10 (*P* values) are greater than 4.3. The variance component based on the kinship analysis employing 347,765 SNPs was computed using the efficient mixed model analysis (EMMA) (Kang et al. 2008) as implemented in SVS V_8.6.0. Narrow sense heritability (pseudo-heritability) was estimated as

$${\text{ph}} = \frac{{\widehat{{\sigma_{\text{g}}^{2} }}}}{{{\text{Var}}(y)}} = \frac{1}{{(1 + \hat{\delta })}},$$

where $$\widehat{{\sigma_{\text{g}}^{2} }}$$ is the estimated genetic variance, Var(*y*) is variance of observed phenotypes, $$\widehat{{\sigma_{\text{e}}^{2} }}$$ is the estimated residual variance, $$\hat{\delta }$$ is $${\raise0.7ex\hbox{${\widehat{{\sigma_{\text{e}}^{2} }}}$} \!\mathord{\left/ {\vphantom {{\widehat{{\sigma_{\text{e}}^{2} }}} {\widehat{{\sigma_{\text{g}}^{2} }}}}}\right.\kern-0pt} \!\lower0.7ex\hbox{${\widehat{{\sigma_{\text{g}}^{2} }}}$}}.$$

Genes co-localized with associated SNPs were identified from the maize GDB genome browser (http://www.maizegdb.org) annotations were obtained from http://ensembl.gramene.org/Zea_mays.

### Validation in bi-parental populations

SNPs found to be significantly associated with kernel-Zn and Fe were selected for single marker QTL analysis in three DH bi-parental populations. In addition, SNPs within the bottom 0.1 percentile of the distribution in the GWAS (Sehgal et al. 2017) were also tested independently for possible linkage to kernel-Zn and Fe concentrations in the DH populations phenotyped at two environments. Single-marker QTL analysis was carried out on single location data, from both the locations obtained from each DH population separately using Gen Stat 14.0.

## Results

### Phenotypic data analysis

Soil characteristic and composition were slightly different between samples from Agua Fria (AF) and Celaya (CE). Soil from CE was of clay type with a pH of 8.03, Zn content of 2.32 µg/g dry weight (DW), Fe 7.84 µg/g DW and N 6.15 µg/g DW. AF soil was of clay loam type with pH of 8.28, Zn content of 1.24 µg/g DW, Fe 17.2 µg/g DW and N 7.57 µg/g DW. ANOVA and other descriptive statistics for both the traits showed significant variability for kernel-Zn and-Fe concentrations among the genotypes of the association mapping panel (Table 1). The average kernel-Zn in the panel was 27.04 µg/g DW, with a range of 17.11–43.69 µg/g DW. The average Fe concentration was 14.65 µg/g DW with a range of 8.19–25.65 µg/g DW. None of the lines in the association mapping panel met the target kernel-Fe concentration of 52 µg/g. Correlations between environments were highly significant for both kernel-Zn and Fe (Table S1). Highly significant, but moderate positive correlation was found between the two traits across the environments (*r* = 0.49, *P* value ≤ 0.001). In the GWAS panel, there were 57 lines (Table S2) which had Zn concentration of 33 µg/g DW or more, the current breeding target for HarvestPlus. Among those 57, there are several elite CIMMYT maize lines (CMLs), 4 QPM lines, several lines susceptible or tolerant to drought, heat or a combination of drought and heat as well as lines susceptible or tolerant to low nitrogen (low N).

**Table 1:** Estimates of mean, variance components (across 3 locations) and broad-sense heritability for kernel-Zn and Fe concentrations in GWAS panel

Trait	Environments	Zn (µg/g)	Fe (µg/g)
Mean	AF12A	26.28 ± 2.61	16.29 ± 1.81
	CE12B	25.26 ± 2.36	13.18 ± 1.68
	AF13A	29.6 ± 3.22	14.48 ± 2.04
	Across	27.04 ± 2.76	14.65 ± 1.85
Range	AF12A	15.20–42.50	8.25–28.60
	CE12B	14.76–50.15	5.65–25.19
	AF13A	17.10–39.80	7.12–27.60
	Across	17.11–43.69	8.19–25.65
LSD	AF12A	5.11	3.54
	CE12B	4.62	3.29
	AF13A	6.30	3.99
	Across	5.41	3.63
CV	AF12A	9.91	11.10
	CE12B	9.34	12.73
	AF13A	10.87	14.07
	Across	10.22	12.64
H^2^	AF12A	0.78	0.83
	CE12B	0.83	0.78
	AF13A	0.76	0.77
	Across	0.86	0.83
*σ* ^2^ *G*		1.28***	4.65***
*σ* ^2^ *GE*		2.12***	10.84***

*AF12A* Agua Fria 2012, *CE12B* Celaya 2012, *AF13A* Agua Fria 2013, *H*^*2*^ broad-sense heritability, *LSD* least significant difference, *CV* coefficient of variance, *G* genotype, *E* environment

***Indicates significance at 0.001

### Principal component analysis and genome-wide linkage disequilibrium

Principal component analysis using genome-wide markers revealed only a moderate population structure with the first three principal components (Fig. S1). The temperate lines and the drought tolerant donor germplasm including both La Posta Sequia and drought tolerant population (DTP) groups clearly separated in different axes from the rest of the CIMMYT tropical and sub-tropical lines. The first three principal components explained 41.86% of the total variance. The genome-wide LD decay was plotted as LD (*r*^2^) between adjacent pairs of markers versus distance in kb between adjacent pairs, and showed that the average LD decay was 17.5 kb at *r*^2^ = 0.1 and 5.99 kb at *r*^2^ = 0.2 (Fig. S2). Chromosome-wise LD analyses showed the slowest LD decay on chromosome 8 (26.54 kb, *r*^2^ = 0.1), followed by chromosome 1 (21.88 kb, *r*^2^ = 0.1).

### GWAS for kernel-Zn and Fe

**Fig. 1:**
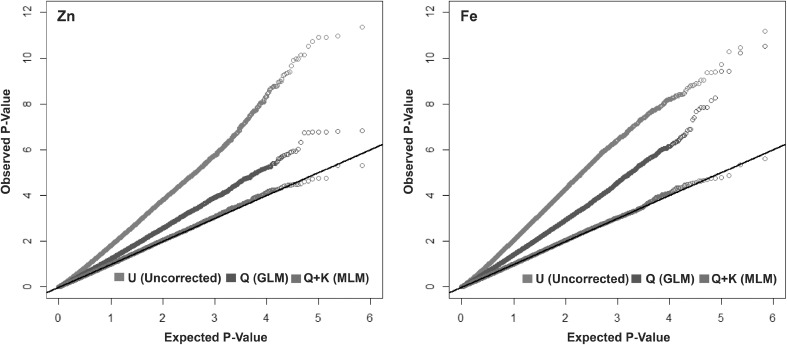
Quantile–quantile (*Q*–*Q*) plots showing inflation of estimated − log10 (*P*) values versus observed for traits Zn and Fe using uncorrected association model (*U*), *Q* (GLM) and *Q* + *K* (MLM). *Q* ten principal components (fixed), *K* kinship matrix (random), *GLM* general linear model, *MLM* mixed linear model

**Fig. 2:**
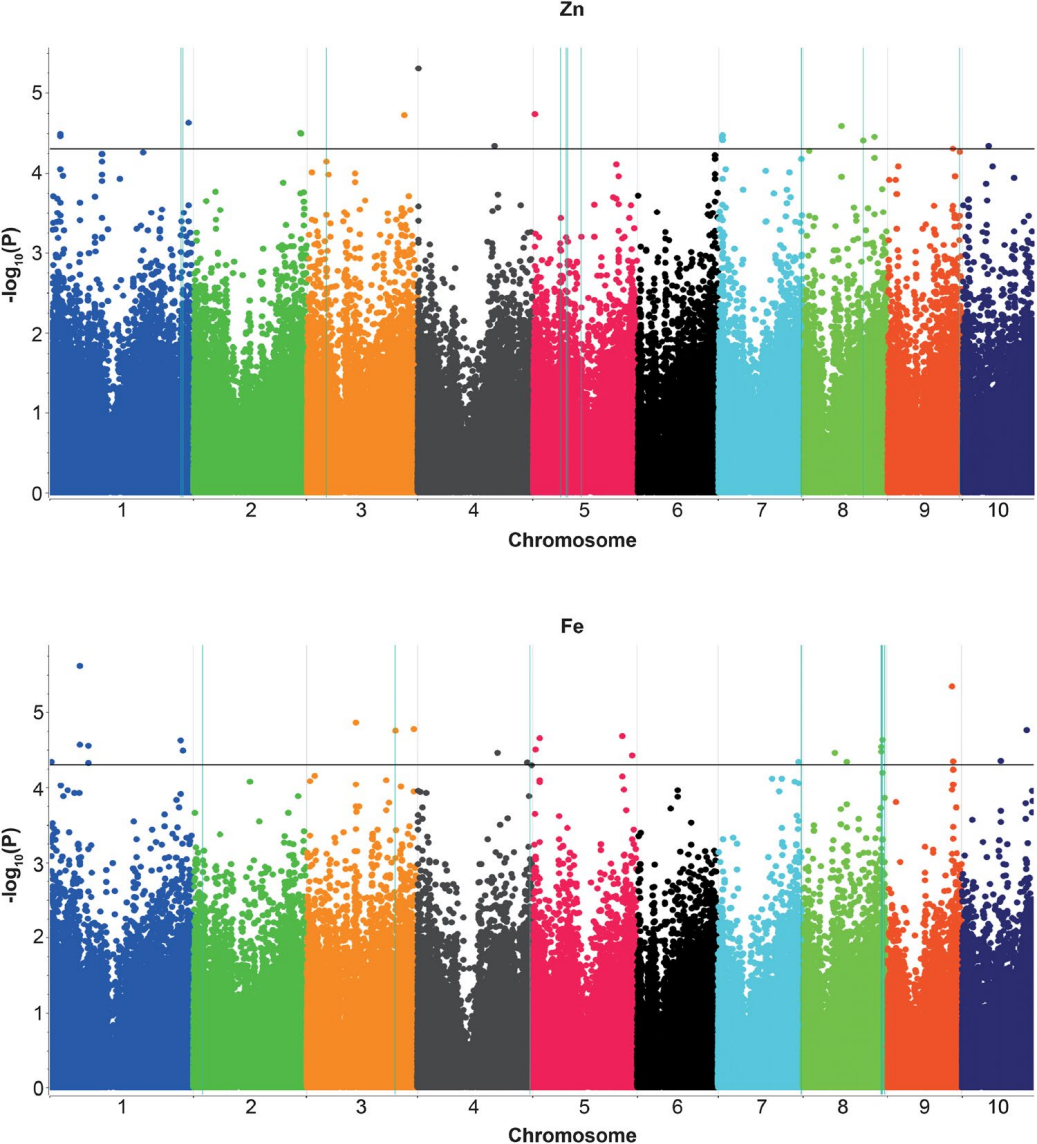
Manhattan plot from the *Q* + *K* (MLM) model for Zn and Fe, plotted with the individual SNPs of all chromosomes on the *X*-axis and − log10 *P* value of each SNP in the *Y*-axis. The different colors indicate the 10 chromosomes of maize. The black horizontal line shows the multiple testing threshold − log10 *P* value of 4.3 for this panel. The blue vertical lines show the associated SNPs validated in bi-parental populations

GWAS was carried out with a subset of SNPs with call rate (CR) more than 0.7 and minor allele frequency (MAF) more than 0.03. 347,765 SNPs which formed this subset were tested against kernel-Zn and Fe concentrations from 923 inbred lines across environments. In the three models used for GWAS, SNPs were tested independently against the phenotypes for Zn and Fe. The uncorrected method (*U*) and the method corrected only for population structure (*Q*) showed significant genomic inflation as observed in the *Q*–*Q* plots (Fig. 1). The *Q* + *K* MLM, where individual SNPs were tested independently correcting for both population structure and kinship, showed the least genomic inflation for both Zn and Fe, and hence significant associations were finalized based on this analysis (Fig. 1). The narrow sense heritability for kernel-Zn was estimated as 0.72 based on the IBS kinship matrix employing all SNPs used in GWAS, with a standard error of 0.16. A total of 20 SNPs were found to have a significant association with kernel-Zn with the *P* value range from 4.93 × 10^−06^ to 5.03 × 10^−05^ (Fig. 2; Table S3) and S4_843764 and S4_843777 on chromosome 4 were found to be the most significantly associated SNPs to kernel-Zn in the panel. Among the 20 SNPs identified for kernel-Zn, 14 were located within predicted gene models, of which 5 were within models with functional domains generally related to metal ion binding or transport or specifically to Zn ion binding (Table S3). Four SNPs identified from the GWAS were found to be located within previously reported QTL for kernel-Zn in maize (Table 4).

For kernel-Fe, a narrow sense heritability of 0.70 was estimated with a standard error of 0.27. 26 SNPs were found to be significantly associated with kernel-Fe with *P* values ranging between 2.43 × 10^−06^ and 5.03 × 10^−05^ (Fig. 2; Table S3), with the maximum number of SNPs found on chromosome 1 (eight SNPs). S1_64238426 on chromosome 1 and S9_136390177 on chromosome 9 were found to be the most significantly associated SNPs with kernel-Fe in this panel. The proportion of variance explained by individual SNPs ranged from 1.8 to 2.41%. Among the 26 SNPs, 20 SNPs were located within predicted gene models. Seven of the GWAS SNPs were located within QTLs reported for kernel-Fe in maize (Table 4).

A set of 57 lines with the highest kernel-Zn content of > 33 µg/g were compared against the same number of lines which had the least kernel-Zn content in the panel. There was a clear enrichment of the favorable allele in all the Zn-associated SNPs in the 57 lines with high kernel-Zn ranging from 51.02 (S10_54119964) to 90.91% (S2_225529232). Similar analysis with high and low kernel-Fe lines showed an allele enrichment of favorable alleles in the high kernel-Fe lines ranging from 52.31% (S10_136070835) to 93.33% (S1_64238509).

### Validation in bi-parental populations

**Fig. 3:**
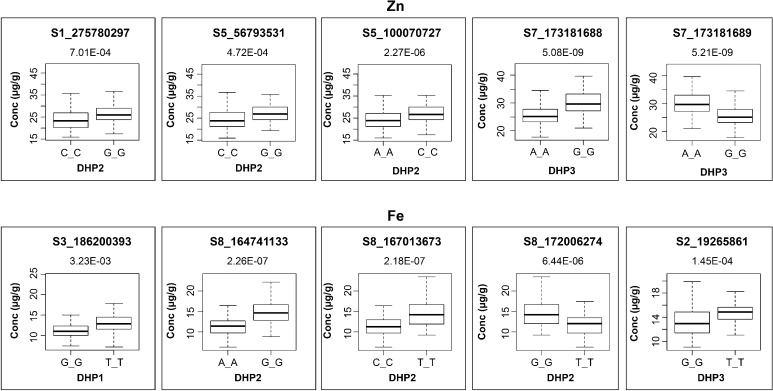
Box plots showing the phenotypic values of the different allele classes of major-effect SNPs validated in DH populations for Zn and Fe (upper and lower panel, respectively). The SNP names, *P* value, alleles and the specific DH population where the effect is witnessed are mentioned near each box. The black horizontal lines in the middle of the boxes are the median values for the Zn or Fe concentration in the respective allele classes. The vertical size of the boxes represents the inter-quantile range. The upper and lower whiskers represent the minimum and maximum values of data

Three DH populations that were phenotyped for kernel-Zn and Fe showed considerable range for the two traits in the two environments studied (Table 2). The kernel-Zn ranged from 15.6 and 48.0 µg/g DW across the two environments and three populations, similarly kernel-Fe ranged between 6.3 and 24.5 µg/g DW. DHP2 showed wider range of concentrations for both kernel-Zn and Fe. Other than the 20 SNPs for kernel-Zn and 26 SNPs for kernel-Fe identified based on GWAS *P* values lower than the panel-determined threshold, 381 SNPs were selected for kernel-Zn and 345 SNPs for kernel-Fe under a reduced threshold limit of *P* value ≤ 1.0 × 10^−03^, for single marker QTL analysis. From these, the polymorphism between the respective parents of each DH population reduced the number of SNPs tested to 232 and 231 SNPs for Zn and Fe, respectively. These SNPs were tested for linkage to kernel-Zn and Fe concentration independently in the three bi-parental populations by analysis of variance due to each allele class in the DH populations. This analysis identified 11 SNPs each for kernel-Zn and Fe that had significant effect on the trait variance (*P* ≤ 0.01, *R*^2^ ≥ 0.05), in one or more populations in one or more environments (Table 3; Fig. 3). Among the 11 SNPs validated for kernel-Zn, six genomic regions could be identified. These were represented by one SNP each on chromosomes 3, 8 and 9, two SNPs each on chromosomes 1 (4 Mb interval) and 7 (adjacent SNPs) and four SNPs on chromosome 5. Among the 11 SNPs validated for kernel-Fe, five broad genomic regions could be identified, with one SNP each from chromosomes 2, 3, 4 and 7, and seven SNPs from chromosome 8, spread in a physical interval of 8 Mb in DHP1 and DHP2. Notable among these were adjacent markers, S7_173181688 (Chr 7: 173,181,688) and S7_173181689 (Chr 7: 173,181,689) that explained 29% (LOD: 9.58) and 28% (LOD: 9.58) of the phenotypic variance for kernel-Zn in DHP3 (Table 3; Fig. 3). Similarly, S8_167013673 (chr 8: 167,013,673) explained 34% phenotypic variance (LOD: 7.72) for kernel-Fe in DHP2, along with two SNPs, S8_164741044 (chr 8: 164,741,044) and S8_164741133 (Chr 8: 164, 74, 1133) which explained about 27% variance (LOD: 7.47) in DHP2 (Table 3; Fig. 3).

**Table 2:** Pedigree and summary statistics of DH populations employed in bi-parental mapping at two locations

DH population	Pedigree	Population size	Trait	Celaya(CE)		Tlaltizapan(TZ)	
				Mean (µg/g)	Range (µg/g)	Mean (µg/g)	Range (µg/g)
DHP1	DTPYC9-F13-2-1-1-2-B-B/CML312	96	Zn	25.61 ± 0.40	17.85–37.45	26.07 ± 0.45	18.50–36.90
			Fe	12.23 ± 0.28	7.10–24.50	15.14 ± 0.35	8.40–22.50
DHP2	CML503/CLWN201	112	Zn	25.32 ± 0.47	15.80–48.00	24.01 ± 0.38	16.15–35.35
			Fe	13.67 ± 0.32	6.30–23.45	12.96 ± 0.30	7.30–22.00
DHP3	CML 465/CML451	143	Zn	27.96 ± 0.39	17.70–43.15	24.08 ± 0.33	15.60–37.80
			Fe	13.91 ± 0.20	9.00–20.70	14.26 ± 0.18	8.20–19.00

**Table 3:** GWAS identified markers validated in DH populations for kernel-Zn and Fe concentration

Trait	SNP	Chr	Physical Position (bp)*	GWAS_*P* value	DHP_*P* value	Add. effect (µg/g)_DHP	ANOVA_adj *R*^2^ (%)	DH population	Effect_location	Gene model	Annotation
Zn	S1_275780297	1	275,780,297	6.12E−04	7.01E−04	− 1.53	10.3	DHP2	CE	GRMZM2G472171	Protein_coding
	S1_279913529	1	279,913,529	5.14E−04	4.78E−03	− 1.57	10.7			GRMZM2G400714	Zinc ion binding and ZOS8-04—C_2_H_2_ zinc finger protein
	S3_40522792	3	40,522,792	7.28E−05	9.63E−03	− 0.82	5.1	DHP3	TZ	GRMZM2G089959	PPR repeat containing protein
	S5_56793531	5	56,793,531	9.09E−04	4.72E−04	1.64	10.7	DHP2	CE and TZ	GRMZM2G116586	UTP-glucose-1-phosphate uridylyltransferase
	S5_68423957	5	68,423,957	6.45E−04	2.79E−03	1.38	7.9			GRMZM2G074896	Glycosyltransferase protein
	S5_71718466	5	71,718,466	7.35E−04	4.49E−06	1.81	13.1			GRMZM2G144282	Translation initiation factor eIF3 subunit
	S5_100070727	5	100,070,727	6.40E−04	2.27E−06	1.60	13.3			GRMZM2G079653	Ice binding, Homoiothermy,
	S7_173181688	7	173,181,688	5.44E−04	5.08E−09	− 2.24	29.2	DHP3		GRMZM2G158162	Zinc ion binding and B3 DNA binding domain containing protein
	S7_173181689	7	173,181,689	6.75E−05	5.21E−09	− 2.20	28.4				
	S8_125472630	8	125,472,630	3.95E−05	1.00E−02	1.33	7.4	DHP2	CE	GRMZM2G048200	GDSL-like lipase,acylhydrolase
	S9_151265550	9	151,265,550	5.51E−05	1.68E−03	1.13	6.6	DHP3		GRMZM2G178775	Putative ribonuclease P
Fe	S2_19265861	2	19,265,861	9.37E−04	1.45E−04	− 0.78	11.0	DHP3	CE	GRMZM2G162333	Pectinesterase activity
	S3_186200393	3	186,200,393	1.78E−05	3.23E−03	− 0.88	10.1	DHP1		GRMZM2G312201	Regulation of transcription and pentatricopeptide
	S4_236412442	4	236,412,442	9.06E−04	2.23E−04	− 1.14	13.9	DHP2	TZ	GRMZM2G471479	–
	S7_174289806	7	174,289,806	8.56E−04	1.97E−03	− 0.90	11.0	DHP1	CE	GRMZM2G130950	5\′-AMP-activated protein kinase beta-2 subunit protein
	S8_164741044	8	164,741,044	2.89E−05	4.03E−04	1.69	27.4	DHP2		GRMZM2G472991	Serine/threonine-protein kinase
	S8_164741133	8	164,741,133	3.38E−05	2.26E−07	1.71	27.7				
	S8_164750150	8	164,750,150	6.59E−04	1.01E−04	1.19	14.8			GRMZM2G172032	Isoprenoid biosynthetic process
	S8_167013673	8	167,013,673	9.99E−04	2.18E−07	1.80	34.1			GRMZM2G074462	Starch binding domain containing protein
	S8_167086477	8	167,086,477	2.36E−05	4.01E−04	1.14	12.6			GRMZM2G162329	nsp1-like C-terminal region family protein
	S8_167089929	8	167,089,929	4.97E−04	6.34E−04	− 1.15	12.9			GRMZM2G061043	nsp1-like C-terminal region family protein
	S8_172006274	8	172,006,274	1.38E−04	6.44E−06	1.47	19.2			GRMZM2G004690	Protein_coding

The chromosomal locations along with effect estimates in different bi-parental populations, along with predicted gene models (B73 AGPV2) within which the SNPs were located are depicted

*Physical positions were retrieved from maize B73 AGPV2. CE and TZ represent the two environments Celaya and Tlaltizapan respectively

## Discussion

### Kernel-Zn and Fe in the mapping panel

In most parts of the maize-growing areas, soils may have different chemical and physical characteristics that can significantly reduce the availability of Zn to plant roots (Cakmak 2008). Hence, the objective of the bio-fortification breeding programs is to develop cultivars that express maximum possible genetic potential to absorb sufficient Zn from the soil and accumulate it in the grain. Zinc availability is highly dependent on pH. Both the soils at CE and AF were slightly alkaline which usually leads to very low Zn availability. Nevertheless, there were lines with Zn concentration as high as 43.7 µg/g DW identified in the mapping panel, and 57 lines had kernel-Zn concentration above the required target. The genotypic variability was also high for the traits making this an ideal population set to study these traits. Among the 57 lines that have higher concentration of Zn compared to the current breeding target, 6 are CMLs (elite CIMMYT maize lines) including CML166, CML192, CML264, CML323, CML361, CML421, which have already been used in hybrid varieties, and no information was previously known about their nutrition content. Interestingly, two of the lines including an elite CML (CML361) are also acid tolerant lines. Among the mechanisms of alleviating aluminum (Al) toxicity, chemical exudates including organic acids, phenolic compounds and phosphates prevent Al from entering to the roots and accumulating in cells (Panda et al. 2009). Some of these mechanisms are common to mineral uptake processes, and could affect Zn uptake from soil.

Four lines identified with high kernel-Zn concentration are QPM (Table S2). An above-average concentration of kernel-Zn was reported in the QPM germplasm as compared to non-QPM/normal maize germplasm (Chakraborti et al. 2009, 2011). In QPM, the presence of opaque-2 allele partially inhibits zein synthesis, with proportional increase in other protein fractions like glutelins, albumins, globulins, proteins known to bind Zn in the endosperm of maize (Diez-Altares and Bornemisza 1967). In addition, Zn plays an important role in tryptophan biosynthesis, which is increased in QPM. In fact, as a part of the breeding program for high Zn at CIMMYT, most of the high zinc germplasm identified so far is QPM, although not all QPM germplasm is high in Zn (Palacios-Rojas, unpublished). Out of the 923 lines used in this study, only 31 were QPM or had QPM background and 33.3% had Zn values higher than 30 µg/g DW. In contrast, out of the 892 non-QPM used in the panel, 19.9% had values higher than 30 µg/g DW, and about 6% of them had values higher than the breeding target (33 µg/g DW). Taken together, these results indicate great potential to develop high Zn maize alone or in combination with better protein quality in bio-fortification programs.

Genetic control for abiotic stresses like drought, heat and a combination of these stresses are largely independent (Cairns et al. 2013) and metabolite responses have highlighted the importance of photorespiration and raffinose family oligosaccharide metabolism (Obata et al. 2015). Under drought and combined drought and heat stress, tryptophan accumulation in maize leaves has been reported in the susceptible genotypes (Obata et al. 2015; Witt et al. 2012). Among the lines with higher Zn concentration, ten genotypes were susceptible to drought or to combined drought and heat stress (Table S2), which is in accordance to the role that Zn plays during tryptophan biosynthesis. However, one genotype tolerant for drought and two tolerant for combined drought and heat stress also had high values of Zn, which underlines the involvement of Zn in other pathways and provides opportunities to develop high Zn germplasm that could be tolerant to such abiotic stresses. Interestingly, four genotypes with high Zn values have been previously reported as susceptible to low-N. It will be important to screen the kernel-Zn accumulation in germplasm exposed to abiotic conditions like drought, heat, low-N, low phosphorous and combined stresses. There is a need to assess if there has been selection against traits like Zn, or if stress tolerance and kernel-Zn can be combined—this is particularly important as drought, heat and combination of stresses are going to become increasingly prevalent under climate change in many countries where mineral deficiency in the diet is prevalent. Equally important is to understand kernel-Zn accumulation in acid-soil tolerant germplasm. This could open the possibility to develop germplasm tolerant to acid soil and capable to accumulate high Zn, which could be ideal products for HarvestPlus target countries like Colombia.

The kernel-Zn and Fe showed significant, but moderate positive correlation in the association mapping panel (*r* = 0.49, *P* ≤ 0.001), which was similar to some earlier reports (Maziya-Dixon et al. 2000; Lung’aho et al. 2011; Baxter et al. 2013) in maize. A significant correlation between grain Zn and Fe concentrations was also reported in wheat (Velu et al. 2011), rice (Kabir et al. 2003), pearl millet (Velu et al. 2008; Gupta et al. 2009) and sorghum (Kumar et al. 2009). This suggests that these traits might have some common genetic mechanisms leading to their accumulation in grains. For instance, some common members of the ZIP family, which is involved in the transport of Zn and Fe as well as of other varieties of divalent cations have been reported (Lee et al. 2010). In addition, several genes responsible for metal chelation, phytosiderophore biosynthesis, uptake, transport, loading and storage of these minerals have been identified in rice, barley, wheat and maize (Gross et al. 2003; Anuradha et al. 2012; Bashir et al. 2012; Sharma and Chauhan 2008).

### Population structure and linkage disequilibrium in the mapping panel

The panel with 923 inbred lines showed moderate population structure within it, based on the principal component analysis. This panel, as discussed before, had germplasm from other breeding programs apart from CIMMYT’s sub-tropical and tropical germplasm. CIMMYT’s elite germplasm showed less spread along the axes, and this has been observed in other studies also, where association mapping panels were constituted only with CIMMYT sub-tropical and tropical lines. In some studies, the first three PCs, explained only about 20% of the total variation (Rashid et al. 2018). Warburton et al. (2002) also observed that the CIMMYT populations, from which most of the sub-tropical and tropical lines have been derived, had a large amount of diversity within, rather than between source populations. Due to the heterogeneous nature of CIMMYT populations, they suggested that it would be difficult to find well-defined structure within CIMMYT lines. The moderate structure that was observed in the present study panel may be due to the inclusion of multiple sources of germplasm, whether from the temperate breeding pools from South Africa or the drought tolerant donor lines from CIMMYT, like LaPosta Sequia and DTP lines. The macro-structure relationship within the panel and the cryptic relatedness due to kinship could confound association mapping (Yu et al. 2009), and hence the need to be using appropriate models to control spurious associations while conducting GWAS. Linkage disequilibrium (LD) is a measure of non-random association of alleles at two or more loci; faster the LD decay, better is the mapping resolution. We found an average LD decay (*r*^2^ = 0.2) across the whole genome close to 6 kb in the panel. This is in accordance with several previous studies (Yan et al. 2009; Lu et al. 2011; Romay et al. 2013; Zhang et al. 2016) employing tropical and sub-tropical maize. This rapid LD decay in the panel is reflective of the genetic diversity of the tropical/subtropical maize germplasm used in this study. Among the ten chromosomes, chromosome 8 was found to have the slowest LD decay (26.54 kb), and this was observed in several previous studies (Suwarno et al. 2014; Rashid et al. 2018).

### GWAS and validation in bi-parental populations

Unlike linkage mapping, association mapping can explore all the recombination events and mutations in a given population and with a higher resolution (Yu and Buckler 2006). Population structure and cryptic relatedness in the form of kinship can create unexpected LD between unlinked loci across the genome. Many statistical procedures using mixed models that correct for these confounding factors have been developed and used in GWAS to minimize the detection of false positives (Yu et al. 2006; Kang et al. 2008). Similarly, some of the real associations fail to be detected due to lack of statistical power. For this reason, GWAS is widely considered as hypothesis generation step, and the marker-trait associations detected are validated through replication in independent association studies or linkage studies in bi-parental populations, to be considered for further applications. Considering this fact, our study was designed to detect SNPs that are significantly associated with kernel-Zn and Fe through GWAS, and these leads were validated in three independent bi-parental populations.

GWAS was performed using multiple statistical models, and the MLM correction for population structure and kinship was found to control the genomic inflation to the minimum level. Marker-trait associations were declared significant based on significance threshold corrected for multiple testing corrections taking average extent of genome-wide LD into consideration. For validation of SNPs, three DH populations were developed and phenotyped at two environments. We selected a higher number of SNPs for testing in the bi-parental populations by including the bottom 0.1 percentile of the distribution to test if SNPs had a significant effect on the phenotype in specific bi-parental populations.

In total 11 SNPs each for kernel-Zn and Fe (*P* ≤ 0.01 and *R*^2^ ≥ 5%) were found to have a significant effect on these traits in at least one population. However, it should be noted that about one-third of the SNPs that were selected for testing were not polymorphic in any of the parental combinations, limiting the ability of them being tested or validated in the present study. Some of the SNPs that were tested explained large proportion of phenotypic variance in individual bi-parental populations, though these could have been slightly over-estimated in single-marker QTL analysis. SNPs S7_173181688 and S7_173181689, located at physical coordinates chr 7: 173,181,688 and chr 7: 173181689, respectively, were shown to have the largest proportion of variance explained for kernel-Zn in the bi-parental populations studied. These will be further tested in breeding populations for their usefulness in selecting lines with high Zn. Similarly, SNPs on chromosome 8, around 164 and 167 Mb were found to explain a large proportion of variance for kernel-Fe. Considering the SNPs that were tested to be significant in trait expression, approximately five genomic regions, represented by one to many SNPs were identified. A region on chromosome 7 within 1 Mb between 173 and 174 Mb was found to be having significant effect for both kernel-Zn and Fe, and will be closely followed in later studies towards using them as breeding targets in Zn and Fe improvement.

### QTLs and candidate genes

Previous studies have reported QTL mapping and meta-QTL analysis for kernel-Zn and Fe in maize (Lung’aho et al. 2011; Qin et al. 2012; Ŝimić et al. 2012; Baxter et al. 2013; Jin et al. 2013). We compared the genomic positions of these QTLs against the ones detected in this study to determine if any of these fall within reported QTL intervals (Table 4). For kernel-Zn, reported chromosomal bins 3.04 (Qin et al. 2012), 4.06, 5.04, (Jin et al. 2013) and 9.06–07 (Qin et al. 2012; Jin et al. 2013) were found to have significant SNPs for kernel-Zn in this study. Similarly, for kernel-Fe, chromosomal bins, 2.04–07 (Qin et al. 2012; Jin et al. 2013), 3.04–06, 4.06 (Jin et al. 2013), 5.01 (Lung’aho et al. 2011; Baxter et al. 2013) and 8.06 (Ŝimić et al. 2012) were found to have significant SNPs for kernel-Fe detected in this study. There has been conflicting reports on identifying co-localized QTLs for the two traits in accordance to the phenotypic correlation between kernel-Zn and Fe (Qin et al. 2012; Ŝimić et al. 2012; Jin et al. 2013, 2015). In our study, we have observed only limited co-localization of the genomic regions controlling these two traits, like the ones on chromosome 4 (161–167 Mb), 7 (173–174 Mb) and 9 (136 Mb).

**Table 4:** GWAS identified SNPs found within the previously reported QTLs for kernel-Zn and Fe

Trait	GWAS-identified SNPs	Chr	Physical position (bp)^a^	QTL interval	QTL bin	QTL study
Zn	S3_40522792	3	40,522,792	umc1504–umc1386a	3.04	Qin et al. (2012)
Zn	S4_161165956	4	161,165,956	bnlg1621a–dupssr16	4.06	Jin et al. (2013)
Zn	S5_100070727	5	100,070,727	umc1110–bnlg1208	5.04	
Zn	S9_151265550	9	151,265,550	umc1310–bnlg128	9.06–9.07	
Zn	S9_151265550	9	151,265,550	dupssr29–bnlg619	9.07	Qin et al. (2012)
Fe	S2_19265861	2	19,265,861	umc1542–umc1042	2.07	Qin et al. (2012)
Fe	S2_19265861	2	19,265,861	bnlg1690–umc1890	2.04–2.07	Jin et al. (2013)
Fe	S3_186200393	3	186,200,393	mmp144a–umc1266	3.04–3.06	
Fe	S4_167189737	4	167,189,737	bnlg1621a–dupssr16	4.06	
Fe	S5_5104719	5	5,104,719	rz87, RZ87	5.1	Lung’aho et al. (2011) and Baxter et al. (2013)
Fe	S8_164741044	8	164,741,044	ZM0825	8.06	Simic et al. (2012)
Fe	S8_164741133	8	164,741,133			
Fe	S8_164750150	8	164,750,150			

The physical coordinates of the GWAS-identified SNPs and chromosomal bins of markers reported in earlier QTL mapping studies are obtained from B73 AGPV2 and maize GDB

^a^Physical positions were retrieved from maize B73 AGPV2

Some of the marker-trait associations identified in this study were co-located within genes which were previously reported to be linked to Zn and Fe uptake, transport or localization in plants. Among the significant associations detected, only one gene (different SNPs from gene GRMZM2G489070 on chromosome 9) was found to be common for both kernel-Zn and Fe. The Zn-associated SNP S8_80619983 near GRMZM2G311974 gene model possesses No Apical Meristem (NAC) domain transcriptional regulator super family protein. Molecular studies have shown that NAC family transcription factors regulates Fe and Zn remobilization from source organs to developing seeds associated with senescence (Ricachenevsky et al. 2013). A NAC transcription factor was also found to increase grain Fe and Zn content in wheat (Uauy et al. 2006). One of the SNPs associated with kernel-Fe (S5_5104719) was located within the gene model GRMZM2G016756, which has active domains of the transcription factor, phytochrome-interacting factor-4, known to regulate auxin biosynthesis (Franklin et al. 2011). Auxin plays a role in the root morphology in response to Fe availability (Chen et al. 2010; Shen et al. 2015). A recent study has also demonstrated evidence of cross-talk between Zn homeostasis and auxin in Arabidopsis (Rai et al. 2015). Within the gene model GRMZM2G147698, two Fe associated SNPs, S1_64238426 and S1_64238509 were located, and this gene has a myb-like DNA-binding domain that is associated with Fe and Zn transport during nutrient deficiency (Shen et al. 2008). On Chromosome 1, SNPs S1_81549746 and S1_81549744 associated with kernel-Fe are within the gene model GRMZM2G302373, which had glutamine-*s* transferase activity that is involved in stress responses including heavy-metal toxicity and synthesis of phenolic compounds including cinnamic acid (Dixon et al. 2002). S4_167189737 associated with kernel-Fe was within the GRMZM2G168369 gene model related to zinc finger C3HC4 TYPE (RING FINGER) family protein. This gene encodes a cysteine-rich domain of 40–60 residues that coordinates two Zn ions and play a key role in the ubiquitination pathway (Lorick et al. 1999). This family of proteins are weakly up-regulated by Fe deficiency in Arabidopsis, giving an indication that they are linked with Fe homeostasis (Buckhout et al. 2009).

In conclusion, the present study is the first report of a Genome-wide association study (GWAS) using high-density genomic data conducted for detecting marker-trait associations for kernel-Zn and Fe in maize. The study identified about 20 and 26 SNPs, respectively, for kernel-Zn and Fe, respectively. A subset of the marker-trait associations was validated using single marker QTL analysis in three bi-parental populations. Whereas some of the genomic regions identified in this study were novel, others were located in already reported QTL intervals. Some of the identified SNPs were located within many known genes involved in uptake, transport and localization of Fe and Zn in plants. More studies are being carried out to validate the utility of the markers identified in this study in the breeding lines and populations, as a precursor to marker-based breeding for bio-fortification of tropical maize for increased kernel-Zn and Fe contents.

## Electronic supplementary material

Below is the link to the electronic supplementary material.

Supplementary material 1 (PDF 85 kb)

Supplementary material 2 (PDF 136 kb)

Supplementary material 3 (PDF 70 kb)

Fig. S1 Three dimensional (3D) plot representing population structure based on the first three Eigen values of principal components analysis (PCA) of GWAS panel using 69,830 SNPs. The color codes are displayed with figure (PNG 149 kb)

Fig. S2 Linkage disequilibrium (LD) decay plot representing the average genome-wide LD decay of GWAS panel using 34,420 genome-wide SNP markers. The values on the Y-axis represents the squared correlation coefficient r^2^ and the X-axis represents the genetic distance in kilobases (kb) (PNG 1932 kb)
