# Supplementary material for: Identification and validation of genomic regions influencing kernel zinc and iron in maize
Source: Theor Appl Genet. 2018 Mar 24;131(7):1443–57. doi: 10.1007/s00122-018-3089-3 (PMC6004279; doi:10.1007/s00122-018-3089-3)
Supplement: Supplementary file 1 — Supplementary material 1 (PDF 85 kb) [file 122_2018_3089_MOESM1_ESM.pdf]

Theoretical and Applied Genetics

Vemuri Hindu, Natalia Palacios-Rojas, Raman Babu, Willy B.Suwarno, Zerka Rashid, Rayalcheruvu Usha, Saykhedkar Gajanan R, Sudha K Nair*

***Corresponding Authors e-mail Id**: sudha.nair@cgiar.org

Identification and validation of genomic regions influencing kernel Zinc and Iron in maize

Table S1. Phenotypic correlation coefficient between different environments for kernel Zn (above diagonal and Fe (below diagonal) at three locations.

Environment	AF12A	CE12B	AF13A
**AF12A**	1	0.65***	0.68***
**CE12B**	0.62***	1	0.62***
**AF13A**	0.68***	0.54***	1

AF12A- Agua Fria 2012, CE12B- Celaya 2013, AF13A- Agua Fria 2013, *** Indicates significance at 0.001
