# Supplementary material for: Identification and validation of genomic regions influencing kernel zinc and iron in maize
Source: Theor Appl Genet. 2018 Mar 24;131(7):1443–57. doi: 10.1007/s00122-018-3089-3 (PMC6004279; doi:10.1007/s00122-018-3089-3)
Supplement: Supplementary file 2 — Supplementary material 2 (PDF 136 kb) [file 122_2018_3089_MOESM2_ESM.pdf]

Theoretical and Applied Genetics

Vemuri Hindu, Natalia Palacios-Rojas, Raman Babu, Willy B.Suwarno, Zerka Rashid, Rayalcheruvu Usha, Saykhedkar Gajanan R, Sudha K Nair*

***Corresponding Authors e-mail Id**: sudha.nair@cgiar.org

Identification and validation of genomic regions influencing kernel Zinc and Iron in maize

Table S2. Lines with Zn content higher than breeding target (33 µg/g)

S.No	Pedigree	BLUE_Zn (µg/g)	BLUE_Fe (µg/g)	Additional traits*
1	KUIcarotenoidsyn-FS17-3-2-B-B-B-B-B-B-B-B-B	43.68	16.44	
2	SM-189-2	42.92	20.19	
3	(DTPYC9-F134-3-1-B)-B-B-B-B	40.29	18.88	Drought tolerant background
4	CML421	39.57	21.49	
5	CA02402/(24STE-5*24STE-17)-BBBB-###-B-3-B-1-BBB-B-B-B-B-B-B-B-B-B-B	39.41	18.6	
6	[[KILIMAST94A]-30/MSV-03-2-10-B-1-B-B-xP84c1F26-2-2-6-B-3-B]F17-1-2-1-1xP43C9-1-1-1-1-1-BBBB-1-xP84c1F26-2-2-6-B-3-B]-2-2-BxCML486]-1-1-B	38.24	16.4	
7	(CML329/MBRC3AmF25-2-1-1-B-B-B)-B-B-B-B	38.22	14.97	
8	CML361	37.95	18.3	Acid soil tolerant
9	SM-189-67	36.77	25.65	Susceptible to Low N
10	SM-189-22	36.74	17.73	
11	CML192	36.39	15.62	QPM
12	(AMATLC0HS71-1-1-2-1-1-1-BBBB-B-B-B)-B-B-B-B	36.28	19.7	Acid soil tolerant background
13	SM-189-23	36.19	17.25	
14	CML166	36.04	17.18	QPM
15	DTPYC9-F38-4-3-1-1-B	35.88	15.02	Susceptible to drought
16	DTPWC9-F18-1-3-1-2-B-B-B-1	35.82	16.28	Susceptible to drought+ heat and to heat

S.No	Pedigree	BLUE_Zn (µg/g)	BLUE_Fe (µg/g)	Additional traits*
17	(MAS[206/312]-23-2-1-1-B-B-B/[BETASYN]BC1-6-1-1-1-1)-B-B-B-B	35.81	17.86	
18	MBRC6BcF280-2-B-#-1-1-B-B-B-B-B-B-B	35.65	22.28	Susceptible to drought, heat and drought+ heat
19	(CML445/[BETASYN]BC1-9-2-3-1-1)-B-B-B	35.6	16.86	
20	RO452W	35.42	16.59	QPM
21	DTPWC9-F18-1-3-1-1-B	35.4	19.59	Tolerant to Low N
22	CKL05019	35.29	14.83	Susceptible to Low N
23	CNO7/8-178	35.26	17.45	
24	(TX03-1035-55-B)-B-B-B-B	35.24	21.09	
25	CNO7/8-258	35.17	14.94	
26	[CML144/[CML144/CML395]F2-8sx]-1-2-3-2-B*5-1	35.14	17.53	Susceptible to drought, heat and drought+ heat
27	CLYN261	35.01	12.62	
28	(KUIcarotenoidsyn-FS17-3-2-B-B-B/(KU1409/DE3/KU1409)S2-18-2-B)-B-1(MAS:L4H1)-2-B-B-B-B	34.88	17.68	
29	BO163W	34.67	14	QPM
30	DTPWC9-F115-1-4-1-1-B-B-B-B	34.65	17.07	
31	([CML226x[CATETODC1276/7619]]F2-25-1-B*7/CarotenoidSyn3-FS8-4-2-B-B//KUIcarotenoidsyn-FS11-1-1-B-B-B)/([CML226x[CATETODC1276/7619]]F2-25-1-B*7/CarotenoidSyn3-FS8-4-2-B-B//CML300)-2-3-B-B	34.65	19.4	
32	(KUIcarotenoidsyn-FS11-1-1-B-B-B/(KU1409/DE3/KU1409)S2-18-2-B)-B-1(MAS:L4H1)-1-B-B-B-B	34.64	16.53	
33	(CML451//CML451/CML300)/(CML451/CML300//CML297)-16-1-B-B-B	34.58	15.93	

S.No	Pedigree	BLUE_Zn (µg/g)	BLUE_Fe (µg/g)	Additional traits*
34	NC486/(NC486/KUIcarotenoidsyn-FS17-3-2-B-B//KUIcarotenoidsyn-FS11-1-1-B-B-B)-14-3-B-B-B	34.53	22.28	
35	P147-#136-5-1-B-1-BBB-B	34.52	19.97	Tolerant to Drought+heat
36	DTPYC9-F13-2-1-1-2-B-B-1	34.46	20.29	Tolerant to Drought+heat
37	(ZM305/[BETASYN]BC1)-29-3-4-B-B-B-B-B-B-B-B	34.43	14.69	
38	(MIRTC4AmF17-B-2-1-B-B)-B-B-B-B	34.4	15.89	
39	(CarotenoidSyn3-FS5-1-5-B-B/CML353//CML486)-6-1-B-B-B-B	34.38	18.98	
40	(KUI3-B-B-B-B)-B-B-B-B	34.32	19.75	
41	[[GQL5/[GQL5/CML202]F2-1sx]-3-1-2-B/[BETASYN]BC1-2-3-1/KUI+SC55SYN#]-B-B-B-12-B-B-B	33.93	13.93	QPM background
42	([ZM305/BETASYN]BC2-273-1-1-B-B)-B-B-B-B	33.86	12.85	
43	[CML226x[CATETODC1276/7619]]F2-25-1-B*7-B/([CML226x[CATETODC1276/7619]]F2-25-1-B*7/KUIcarotenoidsyn-FS17-3-3-B-B//CML300)-2-1-B-B-B	33.83	22.11	
44	SM-189-31	33.65	16.43	
45	[200-7xMAIZSANANDRES-F1-B-xP84c1F26-2-2-4-B-2-B]F93-3-2-1-3xP43C9-1-1-1-1-1-BBBB-1-xP84c1F26-2-2-6-B-3-B]-1-1-BxCML486]-1-1-B	33.61	14.49	Susceptible to drought and to Low N
46	(SAM4(Angola)/[BETASYN]BC1-56-1-1-B-B-B)-B-B-B-B	33.6	18.59	
47	CNO7/8-156	33.57	17.14	
48	[ZM305/BETASYN]BC2-133-1-2-B-B-B-B-B-B-B-B-B	33.48	16.27	
49	KUIcarotenoidsyn-FS11-1-1-B-B-B-B-B-B-B-B-B	33.45	18.64	
50	DTPYC9-F38-4-3-1-2-B-B-B-B	33.32	16.35	Tolerant to drought, susceptible to heat and DT+H
51	(CML451//CML451/CML305)/(CML451/CML305//CML300)-1-1-B-B-B	33.31	18.02	
52	VL05552	33.29	17.13	Tolerant to Low N

S.No	Pedigree	BLUE_Zn (µg/g)	BLUE_Fe (µg/g)	Additional traits*
53	(CML510//CML510/DTPYC9-F69-3-1-1-1-B-B-B-B)/(CML510/DTPYC9-F69-3-1-1-1-B-B-B-B//CML297)-2-1-B-B-B	33.24	16.85	Drought tolerant background
54	[CLQ06901xB113]-F2)-01-05-K1-B-B-B	33.12	13.9	QPM background
55	CL-RCW87=(CL-04321xCML401)-B-19-2-1-BB-B-B	33.04	20.4	Tolerant to heat, susceptible to drought and drought+heat
56	VL054178	33.03	14.24	
57	SM-189-56	33.03	17.01	Susceptible to Low N

* (Cairns et al. 2013) ; CIMMYT maize lines catalog (www.CIMMYT.org); Das et al. 2017 (submitted), information from breeders and physiologists at CIMMYT.
