# Supplementary material for: Identification and validation of genomic regions influencing kernel zinc and iron in maize
Source: Theor Appl Genet. 2018 Mar 24;131(7):1443–57. doi: 10.1007/s00122-018-3089-3 (PMC6004279; doi:10.1007/s00122-018-3089-3)
Supplement: Supplementary file 3 — Supplementary material 3 (PDF 70 kb) [file 122_2018_3089_MOESM3_ESM.pdf]

Theoretical and Applied Genetics

Vemuri Hindu, Natalia Palacios-Rojas, Raman Babu, Willy B.Suwarno, Zerka Rashid, Rayalcheruvu Usha, Saykhedkar Gajanan R, Sudha K Nair*

***Corresponding Authors e-mail Id**: sudha.nair@cgiar.org

Identification and validation of genomic regions influencing kernel Zinc and Iron in maize

Table S3. Significantly associated SNPs for kernel Zn and Fe concentration identified in GWAS. Chromosomal locations along with effect sizes and predicted gene models (B73 AGPV2) are depicted.

Trait	SNP	Chr	Physical Position (bp)*	P-Value	R^2 (%)	FA**	FA Frequency	Gene Model	Annotation
Zn	S4_843777	4	843,777	4.93E-06	2.27	C	0.09	GRMZM2G163427	Protein_coding
	S4_843764	4	843,764	4.93E-06	2.27	A	0.09		
	S5_2521368	5	2,521,368	1.84E-05	2.00	A	0.05	GRMZM2G160064	Metal ion binding/metal ion transport/heavy metal-associated domain containing protein
	S5_2521372	5	2,521,372	1.84E-05	2.00	G	0.05		
	S3_204998729	3	204,998,729	1.91E-05	1.99	T	0.29	GRMZM2G035276	nucleoside-triphosphatase activity
	S1_291258791	1	291,258,791	2.38E-05	1.94	C	0.13	GRMZM2G134227	catalytic activity
	S8_80619983	8	80,619,983	2.58E-05	1.93	A	0.09	GRMZM2G311974	Nac (No apical Meristem) domain transcriptional regulator super family protein
	S2_224173983	2	224,173,983	3.19E-05	1.88	T	0.05	GRMZM2G173377	protein_coding
	S2_225529232	2	225,529,232	3.23E-05	1.88	T	0.12	GRMZM2G458075	protein serine/threonine kinases
	S1_22439336	1	22,439,336	3.26E-05	1.88	A	0.10	GRMZM2G077127	hydrolase, alpha/beta fold family domain containing protein
	S7_7277849	7	7,277,849	3.39E-05	1.87	C	0.25	GRMZM2G107309	zinc ion binding/histone deacetylase
	S1_22439335	1	22,439,335	3.48E-05	1.86	G	0.10	GRMZM2G077127	hydrolase, alpha/beta fold family domain containing protein
	S8_149148541	8	149,148,541	3.56E-05	1.86	C	0.43	GRMZM2G075058	protein_coding//lipase class 3 family protein, putative, expressed
	S7_7065627	7	7,065,627	3.58E-05	1.86	A	0.10	GRMZM2G398996	serine-type endopeptidase activity/gibberellin receptor GID1L2
	S7_7277737	7	7,277,737	3.91E-05	1.84	G	0.28	GRMZM2G107309	zinc ion binding/histone deacetylase
	S7_7277776	7	7,277,776	3.91E-05	1.84	A	0.28		

Trait	SNP	Chr	Physical Position (bp)*	P-Value	R^2 (%)	FA**	FA Frequency	Gene Model	Annotation
	S8_125472630	8	125,472,630	3.95E-05	1.84	C	0.13	GRMZM2G048200	GDSL-like lipase/acylhydrolase
	S10_54119964	10	54,119,964	4.62E-05	1.81	G	0.58	GRMZM2G073371	metal ion binding//superoxide dismutase activity
	S4_161165956	4	161,165,956	4.63E-05	1.81	A	0.25	GRMZM2G406737	-
	S9_136389202	9	136,389,202	5.03E-05	1.79	T	0.28	GRMZM2G489070	-
Fe	S1_64238426	1	64,238,426	2.43E-06	2.41	A	0.07	GRMZM2G147698	myb-like DNA-binding domain containing protein
	S9_136390177	9	136,390,177	4.54E-06	2.28	T	0.13	GRMZM2G489070	-
	S3_102989836	3	102,989,836	1.38E-05	2.05	A	0.12	GRMZM5G837123	Expressed protein
	S3_224631400	3	224,631,400	1.68E-05	2.01	A	0.04	GRMZM2G034684	Protein_coding
	S10_136070835	10	136,070,835	1.75E-05	2.01	G	0.95	GRMZM2G152076	-
	S3_186200393	3	186,200,393	1.78E-05	2.00	G	0.05	GRMZM2G312201	pentatricopeptide
	S5_187810664	5	187,810,664	2.09E-05	1.97	G	0.39	GRMZM2G461034	F box and DUF domain containing protein
	S5_14804657	5	14,804,657	2.21E-05	1.96	T	0.89	GRMZM2G010146	ER to Golgi vesicle-mediated transport
	S8_167086477	8	167,086,477	2.36E-05	1.94	A	0.75	GRMZM2G162329	homoiothermy/ structural constituent of cell wall/S-layer
	S1_275647011	1	275,647,011	2.37E-05	1.94	A	0.14	GRMZM2G131629	protein serine/threonine kinase activity
	S1_64238509	1	64,238,509	2.71E-05	1.92	A	0.07	GRMZM2G147698	myb-like DNA-binding domain containing protein
	S1_81549746	1	81,549,746	2.82E-05	1.91	C	0.63	GRMZM2G302373	glutathione S-transferase
	S8_164741044	8	164,741,044	2.89E-05	1.90	C	0.96	GRMZM2G472991	protein serine/threonine kinase activity
	S5_5104719	5	5,104,719	3.16E-05	1.88	C	0.04	GRMZM2G016756	phytochrome-interacting factor 4
	S1_279877918	1	279,877,918	3.24E-05	1.88	A	0.88	AC186902.4_FGT001	-
	S8_164741133	8	164,741,133	3.38E-05	1.87	G	0.97	GRMZM2G472991	protein serine/threonine kinase activity
	S8_67381104	8	67,381,104	3.47E-05	1.87	C	0.77	GRMZM2G027546	ubiquitin-conjugating enzyme domain containing protein
	S4_167189737	4	167,189,737	3.48E-05	1.86	T	0.13	GRMZM2G168369	Zinc finger C3HC4 TYPE(RING FINGER) family protein
	S5_207514452	5	207,514,452	3.77E-05	1.85	T	0.10	GRMZM2G036418	MAG2, putative,
	S10_81182541	10	81,182,541	4.43E-05	1.82	G	0.93	GRMZM2G123410	OsFBX261 - F-box domain containing protein
	S9_139191545	9	139,191,545	4.57E-05	1.81	G	0.19	GRMZM2G305027	Core histone H2A/H2B/H3/H4 domain containing protein

Trait	SNP	Chr	Physical Position (bp)*	P-Value	R^2 (%)	FA**	FA Frequency	Gene Model	Annotation
	S7_168921933	7	168,921,933	4.60E-05	1.81	A	0.07	GRMZM2G026991	G-protein coupled receptor protein signaling pathway
	S8_93112257	8	93,112,257	4.60E-05	1.81	A	0.55	AC206644.4_FGT003	-
	S1_3741991	1	3,741,991	4.64E-05	1.81	T	0.21	GRMZM2G030567	protein_coding
	S4_230884639	4	230,884,639	4.66E-05	1.80	T	0.10	GRMZM2G080056	pectate lyase activity
	S1_81549744	1	81,549,744	4.77E-05	1.80	A	0.63	GRMZM2G302373	glutathione S-transferase

* Physical positions were retrieved from maize B73 AGPV2, **FA- Favourable Allele
