# Supplementary figures and images for: Identification and validation of genomic regions influencing kernel zinc and iron in maize
Source: Theor Appl Genet. 2018 Mar 24;131(7):1443–57. doi: 10.1007/s00122-018-3089-3 (PMC6004279; doi:10.1007/s00122-018-3089-3)

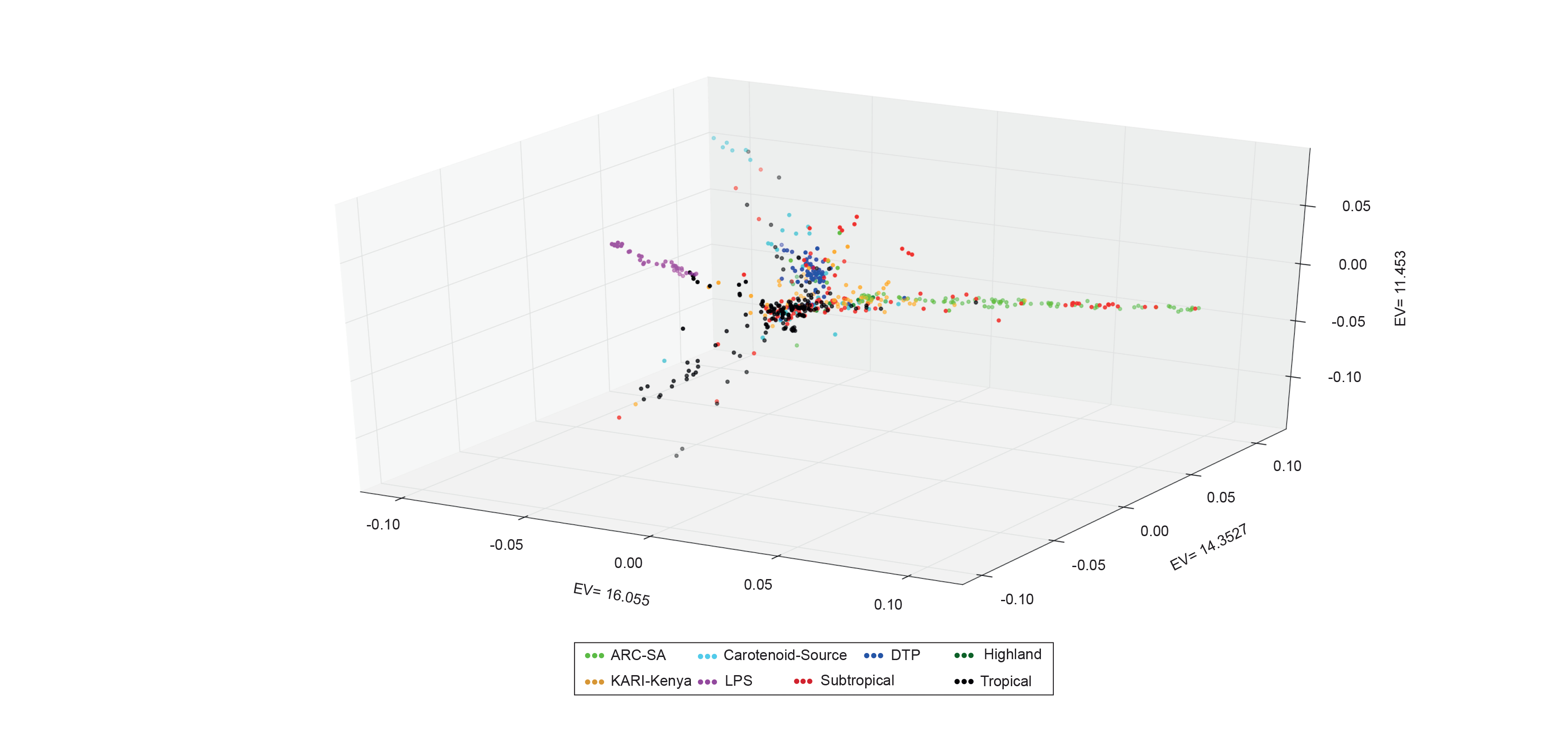

Supplement: Supplementary file 4 — Fig. S1 Three dimensional (3D) plot representing population structure based on the first three Eigen values of principal components analysis (PCA) of GWAS panel using 69,830 SNPs. The color codes are displayed with figure (PNG 149 kb) [file 122_2018_3089_MOESM4_ESM.png]

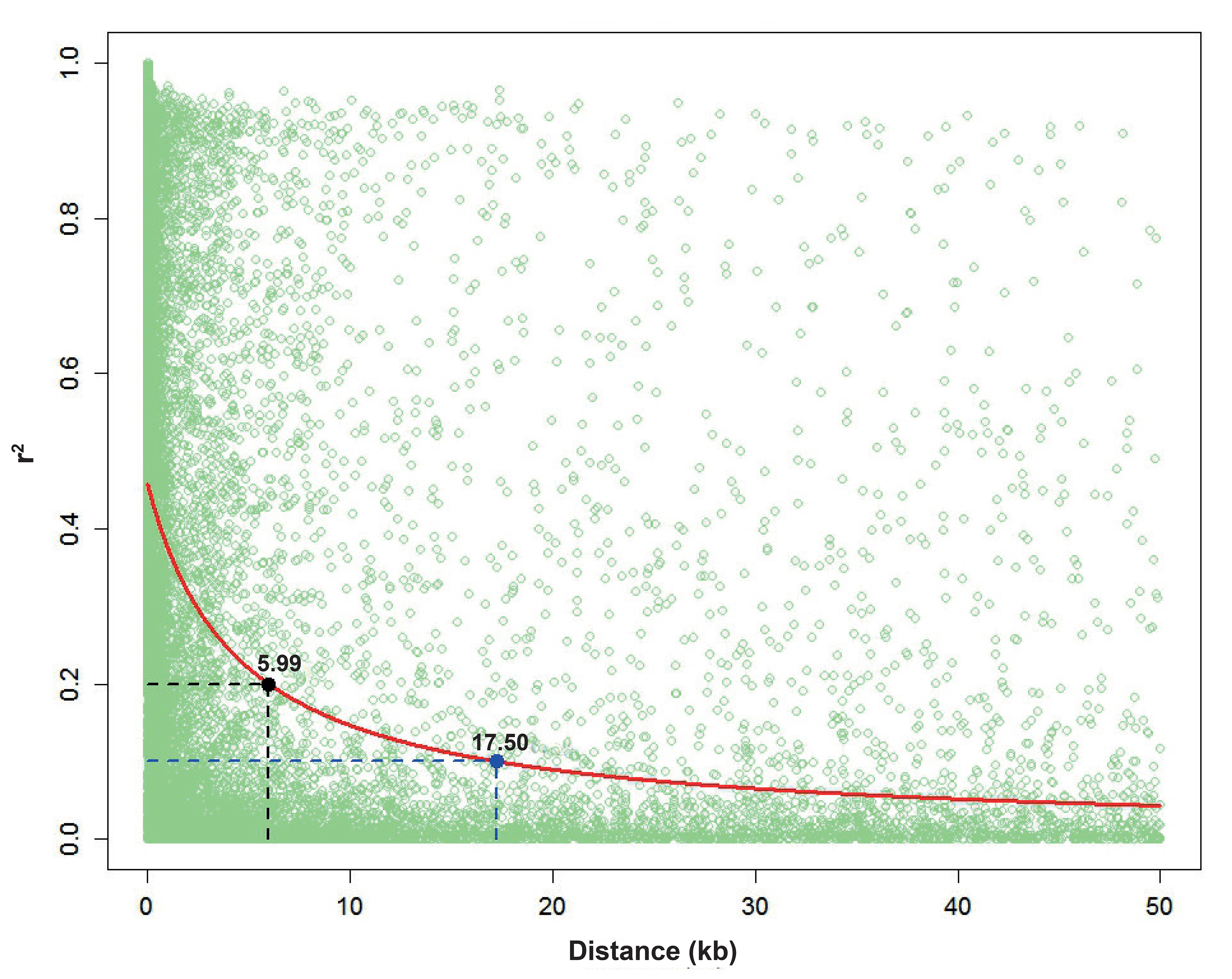

Supplement: Supplementary file 5 — Fig. S2 Linkage disequilibrium (LD) decay plot representing the average genome-wide LD decay of GWAS panel using 34,420 genome-wide SNP markers. The values on the Y-axis represents the squared correlation coefficient r2 and the X-axis represents the genetic distance in kilobases (kb) (PNG 1932 kb) [file 122_2018_3089_MOESM5_ESM.png]
